# Supplementary material for: Epigenetic, ribosomal, and immune dysregulation in paediatric acute-onset neuropsychiatric syndrome
Source: Mol Psychiatry. 2025 Aug 30;30(11):5389–404. doi: 10.1038/s41380-025-03127-5 (PMC12532593; doi:10.1038/s41380-025-03127-5)

Supplementary Table 1

PANS cohort: Clinical features and biological investigations for 36 PANS patients including bulk and 10X single cell RNA sequencing and Toll-like receptor assay

	Age	Gender	Age of onset of symptoms	Pre-existing ASD/ADHD	Pre-existing learning disability/ID	Infection provoked at onset	Acute onset	OCD	Food restriction	Tics	Anxiety	Emotional lability and/or depression	Irritability, aggression, and/or severely oppositional	Behavioural (developmental) regression	Deterioration in school performance	Sensory/motor abnormalities	Somatic signs and symptoms (sleep/urinary disturbance)	PANS Bulk RNA sequencing	10X single cell RNA sequencing	Toll like Receptor assay	PANS-IVIg Bulk RNA sequencing
1	10	M	8															X			
2	13	M	6															X			
3	9	F	7															X			
4	16	F	10															X			
5	9	M	4															X			X #2
6	13	F	9															X			
7	11	M	6															X			
8	10	M	7															X			
9	14	M	10															X			
10	14	M	4															X			
11	11	M	4															X			
12	5	M	3															X			
13	4	F	2															X			
14	11	M	7															X			
15	11	F	9															X			
16	5	M	2															X			
17	4	M	3															X			
18	14	F	10															X			
19	12	F	10															X		X	
20	11	F	4															X			X #1
21	15	F	7																		X #1
22	15	M	14																		X #2
23	10	M	9.5																		X #2
24	14	M	12																		X #2
25	13	F	12																		X #2
26	7	M	6																	X	
27	7	M	4																X	X	X #1
28	9	F	5																X		X #1
29	8	M	3.5																		
30	7	M	5																		
31	15	M	10																		
32	7.8	F	4																		
33	15	M	7																	X	
34	8	M	5																	X	
35	14	F	8																	X	
36	5	F	3																	X	

Supplementary Table 2
Controls: Biological investigations for 29 controls including bulk and 10X single cell RNA sequencing and Toll-like receptor assay
PCOS: polycystic ovarian syndrome

	Condition	PANS- Bulk RNA sequencing	10X single cell RNA sequencing	Toll like receptor assay	PANS IVIg- Bulk RNA sequencing
1	Moderate ID, dup chromosome 5, obesity, workup for glucose intolerance	X			
2	Obesity, workup for insulin resistance	X			
3	Workup for possible PCOS, obesity	X			
4	Delayed puberty	X			
5	Growth delay	X			
6	Previous raised intracranial pressure, now healthy, recovered	X			
7	Epilepsy, cluster of seizures, controlled	X			
8	Workup for possible PCOS	X			
9	Growth failure	X			
10	Workup for possible PCOS	X			
11	Idiopathic generalised epilepsy, controlled	X			
12	Post craniotomy surgery, endocrine assessment	X			
13	Post hypoxic ischemic encephalopathy, spastic quadriplegia and epilepsy, controlled	X			
14	Short stature	X			
15	Intellectual disability, prematurity	X			
16	Precocious puberty		X (10X)		X#1
17	Short stature		X (10X)		X #1
18	Healthy			X	
19	Short stature			X	X #2
20	Healthy			X	
21	Short stature			X	X #2
22	Healthy			X	
23	Precocious puberty			X	X #1
24	Healthy			X	
25	Healthy				X #1
26	Healthy				X #2
27	Healthy				X #2
28	Healthy				X #2
29	Healthy				X #2

Supplementary Table 3

Comparison of PANS versus non-PANS NDD: Clinical features

*Statistical analysis using chi-square test

Clinical features	PANS n=32 (%)	Non-PANS NDD n=68 (%)	p value*
OCD	26 (81)	25 (37)	0.00001
Anxiety	27 (84)	39 (57)	0.001
Depression	22 (69)	5 (7)	0.00001
ASD	12 (38)	24 (35)	0.66
ADHD	9 (28)	32 (47)	0.005
Tourette syndrome/tics	15 (47)	53 (78)	0.00001
Regression in developmental skills	27 (84)	53 (78)	0.28
Triggers at onset			
Infection	21 (66)	5 (7)	0.0001
Stress/others	11 (33)	5 (7)	0.0001
Exacerbating factors of NDDs at any time in clinical course			
Infection	21 (66)	30 (44)	0.001
Stress	25 (78)	56 (82)	0.48
Prolonged school absence (>3 months)	20 (63)	12 (18)	0.00001

Supplementary Table 4
Controls for cerebrospinal fluid (CSF) metabolomics

Autoimmune encephalitis control group	
	Condition
1	Anti-NMDA receptor encephalitis
2	Anti-NMDA receptor encephalitis
3	Anti-NMDA receptor encephalitis
4	Anti-NMDA receptor encephalitis
5	Anti-NMDA receptor encephalitis
6	Anti-NMDA receptor encephalitis
7	Anti-NMDA receptor encephalitis
8	Anti-NMDA receptor encephalitis

Neurogenetic control group	
	Condition
1	Spinocerebellar syndrome
2	GCH1 Segawa disease
3	Neurotransmitter, 6PTPS deficiency
4	Genetic congenital myasthenia
5	TITF1 microdeletion, hereditary chorea
6	GCH1 Segawa disease
7	DYT1 dystonia
8	TNPO2 biallelic variants
9	Suspected neurogenetic
10	NF1, headache
11	Epilepsy, severe neurodisability

Supplementary Text- Case vignettes

Case vignettes of two children with Paediatric acute neuropsychiatric syndrome (PANS) who were selected for single cell RNA sequencing (as Figure 4). Case vignette of one child with PANS who received intravenous immunoglobulin treatment (as Figure 6B).

Case 1

Case 1 is a ten-year old boy who was initially neurodevelopmentally normal but had acute onset change in emotional regulation, memory, and personality at the age of 4. This is in the context of recurrent acute deteriorations resulting in significant functional impairments. He has a significant family history of immune dysregulation. His mother has multiple sclerosis (MS), diagnosed at 20 years of age, and has anxiety since then. In addition, maternal aunt, and maternal granduncle have MS, maternal grandfather has depression. His father has Crohn's disease diagnosed at age 20, paternal uncle has obsessive compulsive disorder. The pregnancy was normal, but his mother took antibiotics due to previous urinary tract infections resulting in premature delivery. There was placental abruption, but he was well at birth. His development was otherwise normal; he was very bright and there was no premorbid anxiety.

He was developmentally normal up till the age of 4, when he experienced abrupt onset change in behaviour and personality. He had bouts of eye blinking for a week on two occasions. A pronounced escalation in emotional symptoms, anxiety, and obsessive symptoms linked to anger was evident, causing notable functional impairments. In response to these challenges, his family sought assistance and presented themselves to the hospital. He would ask his parents repeatedly "are you guys going to remember that?", "did you hear everything", "will you never forget", suggestive of anxiety/obsession and the need for reassurance. He appeared to have memory change, that fluctuate and can include routine activities- eg. forgetting to scan items at shop checkout. These emotional, memory, change in personality fluctuate significantly and are worse with infections. Bad emotional periods were also associated with a new onset urinary incontinence , manifesting as full diapers overnight and diaper leak. Over time, he also had loss in motor function including swimming. His magnetic resonance imaging brain scan, electroencephalogram and blood tests were normal. He was treated with Sertraline and Guanfacine. He experienced transient but not sustained improvements with 6 courses of monthly intravenous immunoglobulin (IVIg), thus IVIg was stopped.

Case 2

Case 2 is a nine-year-old girl who was neurodevelopmentally normal but had acute onset emotional symptoms of anxiety, obsessive compulsive behaviours and regression of school performance with school refusal at the age of 6. Her mother has Graves' disease and psoriasis. Maternal uncle has mature onset diabetes of the young. Maternal grandmother has arthritis and anxiety, parental grandmother has Graves' disease. Her mother had symptoms of depression during pregnancy in addition to significant morning sickness.

Prior to change in function, the child was described as placid and easy-going. She had a distinct change in her behaviour at age 6. One of the most noticeable symptoms was that she could not pick up her violin to practice. She became distressed and overwhelmed when faced with picking it up to practice. At that same time, there was a significant regression in her school performance. She was previously top of her class and a very enthusiastic learner. However, she regressed from writing a page to either refusing to write or writing simple sentences and she scored zero for geography. She also became slow to get dressed In the mornings, and perseverated on a task such as putting on a sock to the extent that she became very anxious that she could not do it. At times, her anxiety was extremely overwhelming and she became 'stuck' in her thinking. This was associated with emotional meltdowns, screaming and "fight and flight" episodes. She also had the tendency to negativity, overthinking, internalisation of concerns and expressed thoughts of suicidality.

She received IVIg with significant improvements in anxiety, emotional regulation and reduction of "intrusive thoughts" around violin. She was also commenced on Sertraline and Intuniv. IVIG was ceased after 6 months, and she has remained well and able to continue with conventional treatment only.

Case 3

Case 3 is a fourteen-year-old girl who had abrupt onset OCD presentation, emotional dysregulation, rage, behavioural regression and decline in school performance, after COVID infection. She has asthma. Her brother has ASD level 1. Her mother has ulcerative colitis since young adulthood, and endometriosis.

At age 13, one week after COVID upper respiratory tract infection, she started to have abrupt onset obsessive compulsive behaviours. She would be in extreme distress, shouting, crying, she would freeze in positions and seemed to be hyperventilating. Sometimes, there were clear compulsive repetitive movements – such as stepping on and off the top steps of the stairs, and repeating to step backwards over and over. There was a major change in her personality, she stopped walking, talking, eating, wet herself and did not go to school for two months. She trialled Lexapro, Seroquel, Luvox, low dose naltrexone, steroids, and antibiotics during this period. There was transient improvement with antibiotics, but OCD symptoms were remitting and relapsing in nature. She would take an hour to get dressed by taking clothes on and off over in her head. She had repetitive and intrusive thoughts of "bad things". She became very sensitive to sounds and environmental stimuli.

Since commencing monthly IVIg treatment, there have been significant improvements in her OCD, anxiety symptoms and function. After 6 months of IVIg therapy, her CY-BOCs score improved from 46/50 (incapacitating OCD) to 25/50 (moderate OCD). She has since been able to attend school daily, able to complete homework, communicate better with family and self-care. Other than IVIg, she was also taking Clomipramine 25mg daily, naltrexone 3mg daily and continued regular psychological therapies. Unfortunately, trial off IVIg for 3 months was associated with relapse of OCD symptoms, and she has since recommenced on IVIg treatment.

Supplementary Methods

Bulk RNA sequencing method

This workflow includes RNA extraction from PAXgene™ blood RNA tubes, depletion of ribosomal RNA via hybrid capture (Illumina Ribo-Zero), and Illumina TruSeq Stranded Total RNA Library Preparation (input 200-1000 ng of Total RNA). The stranded RNA samples are sequenced on the Illumina NovaSeq 6000 next generation sequencing platform (2 x 150 base pair) for a depth of 50 million paired end reads. The cleaned sequence reads were aligned against the homo sapiens genome (Build version hg38), and the STAR aligner (v2.5.3a) was used to map unique reads to the genomic sequences[1].

Single cell RNA sequencing

Peripheral blood mononuclear cells (PBMCs) were isolated from the whole blood within 6 hours of sample collection using a Ficoll density medium (Cytiva) in SepMate tubes (Stemcell Technologies). PBMCs were cryopreserved in 10% dimethyl sulfoxide (Amrescro, WN182-10ML) in foetal bovine serum (FBS, Thermo Fisher Scientific) and stored in liquid nitrogen until analysis with 10X Genomics single cell RNA sequencing.

PBMCs were thawed from liquid nitrogen, stained using DAPI, and sorted into a single cell suspension of live cells. A small aliquot was used to assess viability of the single cell suspensions using 0.4% Trypan Blue staining on a Countess II Automated Cell Counter (Invitrogen). Cell concentrations were adjusted to 1000 cells/µl and cell suspensions were loaded onto a Chromium Next GEM Chip G (10X Genomics) for a target output of 10,000 cells per sample.

Single-cell droplet capture was performed on the Chromium Controller (10X Genomics) by Children's Medical Research Institute single cell sequencing centre. cDNA library preparation was performed in accordance with the Chromium Next GEM Single Cell 3' v3.1 (dual index) protocol. Libraries were evaluated for fragment size and concentration using Agilent HSD5000 ScreenTape System. The sequencing was performed by AZENTA Life Sciences, China on an Illumina HiSeq4000 instrument according to manufacturer's instructions (Illumina). Sequencing was carried out using 2x150 paired end configuration with a sequencing depth of 20,000 reads per cell. Raw sequencing reads were mapped and counted against human reference genome (GRCh38) using 10X Genomics Cell Ranger 6.1.0. Multi-copy reads were discarded and only uniquely mapping reads were carried forward to unique molecular identifier (UMI) counting. The UMI count matrices were imported into the R package *Seurat* in the R statistical environment (v 4.2.3) [2].

Toll-like receptor immune assay

PBMC isolation was performed (by SA, SP, BK) using density gradient centrifugation and samples were stored in liquid nitrogen until thawed (as above). Cells were cultured in Roswell Park Memorial Institute media (RPMI; Cat #11875093, Thermo Fisher), supplemented with 10% fetal bovine serum (FBS) and 1x Glutamax (Cat #35050061, Thermo Fisher) such that there were 500,000 cells/per well in sterile 96-well U-bottom plates. Cells were allowed to rest for 3hours in the incubator prior to any stimulation. Stimulation was performed using 500ng/mL Lipopolysaccharide (LPS, from Escherichia coli 0111:B4; Cat #tlrl-3pelps, InvivoGen) for 30 minutes, 3 hours, and 24 hours in staggered timing. An unstimulated condition was included in duplicates for each sample by adding warm PBS (-/-) as a control. Supernatant was collected for ELISA, while the pellet was resuspended in RLT buffer supplemented with B-mercaptoethanol and stored until RNA extraction was performed.

RNA was extracted using the Rneasy Mini Kit (Cat # 74106, Qiagen) according to manufacturer's instructions. RNA with a RIN >8 and a concentration > 10ng/uL were reverse transcribed to synthesise complementary deoxyribonucleic acid (cDNA) using the SuperScript™ IV Reverse Transcriptase (Cat #18090010, Thermo Fisher) according to manufacturer's instructions. Reverse transcribed quantitative polymerase chain reaction (RT-qPCR) was performed using a QuantStudio 6Pro (Thermo Fisher) in 384-well plates, containing 0.28ng of cDNA. RT-qPCR data was analysed using the delta-delta comparative threshold method. Each PCR contained a housekeeping reference gene for all samples; Beta 2-microglobulin, which was used as the reference in all calculations. The fold changes were imported into GraphPad Prism v8.2.0, where all statistical analyses were performed. Each dataset was tested for normality using a Shapiro-Wilks test and statistical analysis was employed using a Mann-Whitney test.

Cytokine production: Soluble cytokines were measured in the supernatant of samples using human ELISA kits for IL6 and TNF (Cat #EH2IL6, and #KAC1751, Thermo Fisher) according to manufacturer's instructions. Samples were diluted 1:20.

Supplementary methods

Bulk RNA sequencing bioinformatic analysis

For bulk RNA sequencing, filtering and normalization steps were first performed on the dataset. Subsequently, normalization with removal of unwanted variation, via the remove unwanted variation *(RUV)* R package was performed [3]. RUV is commonly used in human samples to better discern biologically relevant variations associated with the disease of interest, particularly in the presence of significant inter-sample biological diversity[4]. This method relies on having a set of endogenous negative control genes known *a priori* not to be differentially expressed with respect to the biological factor of interest [3, 4]. For this study, a set of 500 empirical negative control proteins with little or no change in RNA expression across samples was identified from an initial ANOVA test. In the PANS cohort and the PANS-IVIg cohort, *k=8* and *k=10* (factors of unwanted variation) were used respectively to remove genes that had minimal differential expression, compared to negative control genes.

For linear modelling, the *limma* R package was used and the p-values were calculated using the empirical Bayes method 'eBayes' function[5]. The false discovery rate correction was applied to the p-values by calculating the adjusted p-values. Significant differentially expressed genes were defined as those with adjusted p-values/false discovery rate (FDR) less than 0.05.

Single cell RNA sequencing bioinformatic analysis

Cells with a high mitochondrial transcript ratio (>0.15) were excluded. Experiments were integrated using the *FindIntegrationAnchors* function in *Seurat* then immune celltypes were assigned using *scPred* [6]. Merged data were then split by cell type and separately normalised, scaled, and integrated between patients using *harmony* [7], then UMAP (uniform manifold approximation and *projection)* projections were made using the first 30 dimensions. Differentially expressed genes were identified using *FindMarkers.*

Enrichment analyses

The genes were ranked based on their sign(logFC) x log10Pvalue scores [8, 9]. Enriched gene sets are identified based on a running sum statistic (normalized enrichment score (NES)) and statistical significance, based on the FDR. We performed enrichment analysis on bulk RNA sequencing data for both the first and second (PANS-IVIg) PANS cohorts, as well as on single-cell RNA sequencing data. In the second (PANS-IVIg) PANS cohort, consisting of two batches, we conducted separate enrichment analyses within each batch, as well as combined. The results of the enrichment analysis for individual batches were similar to those obtained when the batches were analyzed together (data not shown).

Bar and dot plots of GSEA results were plotted using *ggplot2* package, and heatmaps of GSEA results were made using the *pheatmap* package. Connectivity network (CNET) plots were created using *enrichplot* package where the enriched pathways are represented by their respective colors, and corresponding genes' adjusted p value.

References

1. Dobin A, Davis CA, Schlesinger F, Drenkow J, Zaleski C, Jha S *et al.* STAR: ultrafast universal RNA-seq aligner. *Bioinformatics* 2013; **29**(1)**:** 15-21.
2. Hao Y, Hao S, Andersen-Nissen E, Mauck WM, 3rd, Zheng S, Butler A *et al.* Integrated analysis of multimodal single-cell data. *Cell* 2021; **184**(13)**:** 3573-3587.e3529.
3. Risso D, Ngai J, Speed TP, Dudoit S. Normalization of RNA-seq data using factor analysis of control genes or samples. *Nat Biotechnol* 2014; **32**(9)**:** 896-902.
4. Gagnon-Bartsch JA, Speed TP. Using control genes to correct for unwanted variation in microarray data. *Biostatistics* 2012; **13**(3)**:** 539-552.
5. Ritchie ME, Phipson B, Wu D, Hu Y, Law CW, Shi W *et al.* limma powers differential expression analyses for RNA-sequencing and microarray studies. *Nucleic Acids Res* 2015; **43**(7)**:** e47.
6. Alquicira-Hernandez J, Sathe A, Ji HP, Nguyen Q, Powell JE. scPred: accurate supervised method for cell-type classification from single-cell RNA-seq data. *Genome Biology* 2019; **20**(1)**:** 264.
7. Korsunsky I, Millard N, Fan J, Slowikowski K, Zhang F, Wei K *et al.* Fast, sensitive and accurate integration of single-cell data with Harmony. *Nat Methods* 2019; **16**(12)**:** 1289-1296.
8. Subramanian A, Tamayo P, Mootha VK, Mukherjee S, Ebert BL, Gillette MA *et al.* Gene set enrichment analysis: a knowledge-based approach for interpreting genome-wide expression profiles. *Proc Natl Acad Sci U S A* 2005; **102**(43)**:** 15545-15550.
9. Reimand J, Isserlin R, Voisin V, Kucera M, Tannus-Lopes C, Rostamianfar A *et al.* Pathway enrichment analysis and visualization of omics data using g:Profiler, GSEA, Cytoscape and EnrichmentMap. *Nature Protocols* 2019; **14**(2)**:** 482-517.

Supplementary Figure 1A: Novel purpose-built Infection screening tool for first five years of life

In the first five years of your child's life, how often were the following symptoms present?					
	Never	Occasional (less than once per year)	Sometimes (1-3 times per year)	Often (4-6 times per year)	Almost always (so frequent, hard to count)
Clear runny nose – first 5 years of life					
Clear runny nose – last 12 months					
Urinary tract infection – first 5 years of life					
Urinary tract infection – last 12 months					
Throat infection/tonsilitis – first 5 years of life					
Throat infection/tonsilitis – last 12 months					
Ear infection with pain or pus – first 5 years of life					
Ear infection with pain or pus – last 12 months					
Sinus infection – first 5 years of life					
Sinus infection – last 12 months					
Pneumonia – first 5 years of life					
Pneumonia – last 12 months					
Mouth ulcers -– first 5 years of life					
Mouth ulcers – last 12 months					
Skin infection (impetigo) – first 5 years of life					
Skin infection (impetigo) – last 12 months					
Meningitis or other serious infection (bone, joint, blood) – first 5 years of life					
Meningitis or other serious infection (bone, joint, blood) – last 12 months					
GP visit for infection – first 5 years of life					
GP visit for infection – last 12 months					
Antibiotic courses – first 5 years of life					
Antibiotic courses – last 12 months					
Emergency department visit for infection – first 5 years of life					
Emergency department visit for infection – last 12 months					
Hospitalisation for infection – first 5 years of life					
Hospitalisation for infection – last 12 months					

Supplementary Figure 1B: Novel purpose-bullt Infection screening tool for last 12 months prior to interview

In the last 12 months of your child's life prior to interview, how often were the following symptoms present?					
	Never	Occasional (less than once per year)	Sometimes (1-3 times per year)	Often (4-6 times per year)	Almost always (so frequent, hard to count)
Clear runny nose – first 5 years of life					
Clear runny nose – last 12 months					
Urinary tract infection – first 5 years of life					
Urinary tract infection – last 12 months					
Throat infection/tonsilitis – first 5 years of life					
Throat infection/tonsilitis – last 12 months					
Ear infection with pain or pus – first 5 years of life					
Ear infection with pain or pus – last 12 months					
Sinus infection – first 5 years of life					
Sinus infection – last 12 months					
Pneumonia – first 5 years of life					
Pneumonia – last 12 months					
Mouth ulcers -– first 5 years of life					
Mouth ulcers – last 12 months					
Skin infection (impetigo) – first 5 years of life					
Skin infection (impetigo) – last 12 months					
Meningitis or other serious infection (bone, joint, blood) – first 5 years of life					
Meningitis or other serious infection (bone, joint, blood) – last 12 months					
GP visit for infection – first 5 years of life					
GP visit for infection – last 12 months					
Antibiotic courses – first 5 years of life					
Antibiotic courses – last 12 months					
Emergency department visit for infection – first 5 years of life					
Emergency department visit for infection – last 12 months					
Hospitalisation for infection – first 5 years of life					
Hospitalisation for infection – last 12 months					

Supplementary Figure 2: Socio-Economic Indexes for Areas (SEIFA) distribution in NDD vs controls.

Dot plot of Socio-Economic Indexes for Areas (SEIFA) distribution in neurodevelopmental disorders (NDDs) group versus healthy controls (y axis represents SEIFA decile). The lowest 10% of areas are given a decile number of 1, and the highest 10% of areas are given a decile number of 10 (1 being poor, and 10 being rich)

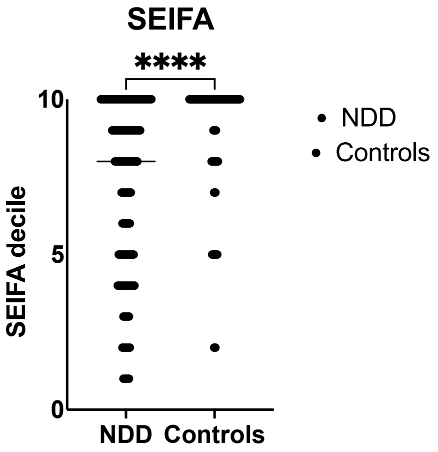

Supplementary Figure 3A:
Infection frequencies in the first 5 years of life in NDD vs healthy controls

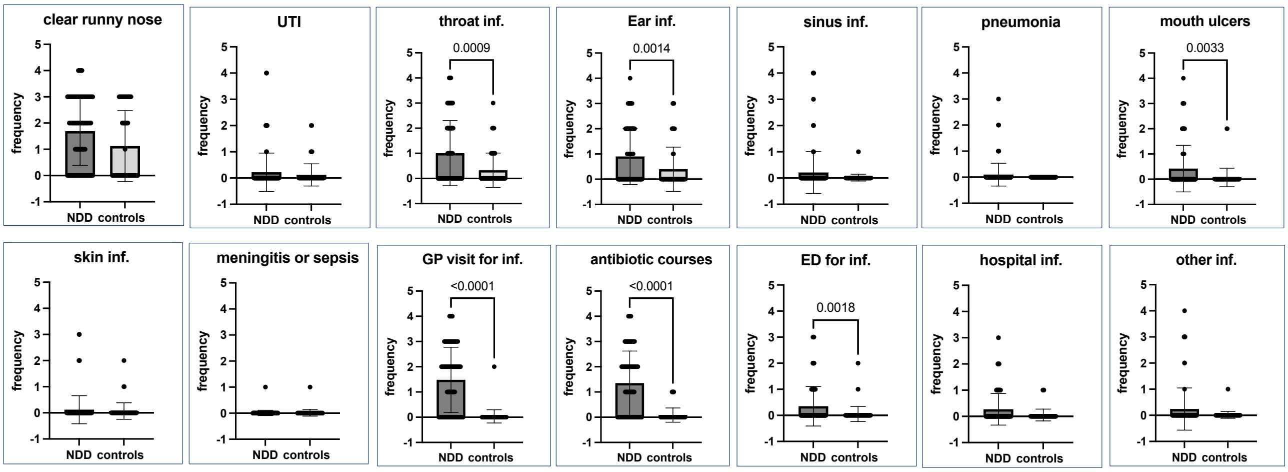

Infection frequencies in the 12 months prior to assessment in NDD vs healthy controls

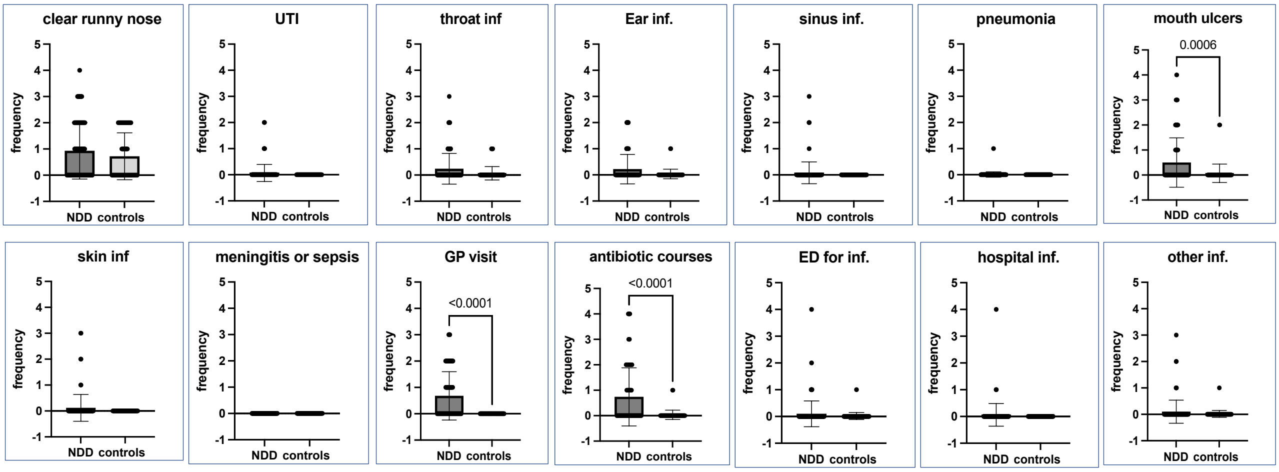

Supplementary Figure 3B: Heatmap of infection frequencies in first 5 years of life and last 12 months prior to interview between PANS and NDD non-PANS patients

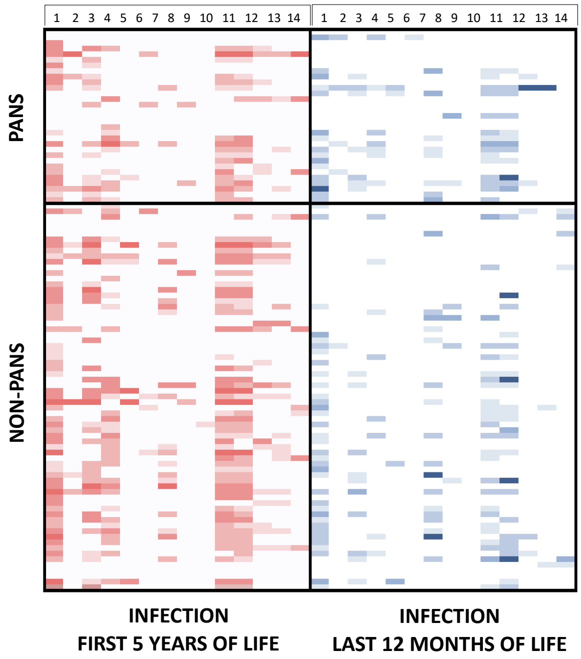

Heatmap of infection frequencies in first 5 years of life and last 12 months of life between patients who fulfil criteria for Paediatric acute neuropsychiatric syndrome (PANS- top half) and those NDD patients who did not (non-PANS- bottom half). On the x axis, numbers 0 to 14 represent the 14 questions in the infection screening tool (as per Supp Figure 1 (1 is 'clear runny nose' and 14 is 'hospitalized for infection'). The intensity of the colour represents the frequency based on the 5-point Likert scale.

Supplementary Figure 4A: Strengths difficulties questionnaire (SDQ) in NDD (n=100) vs healthy controls (n=58)- individual domains

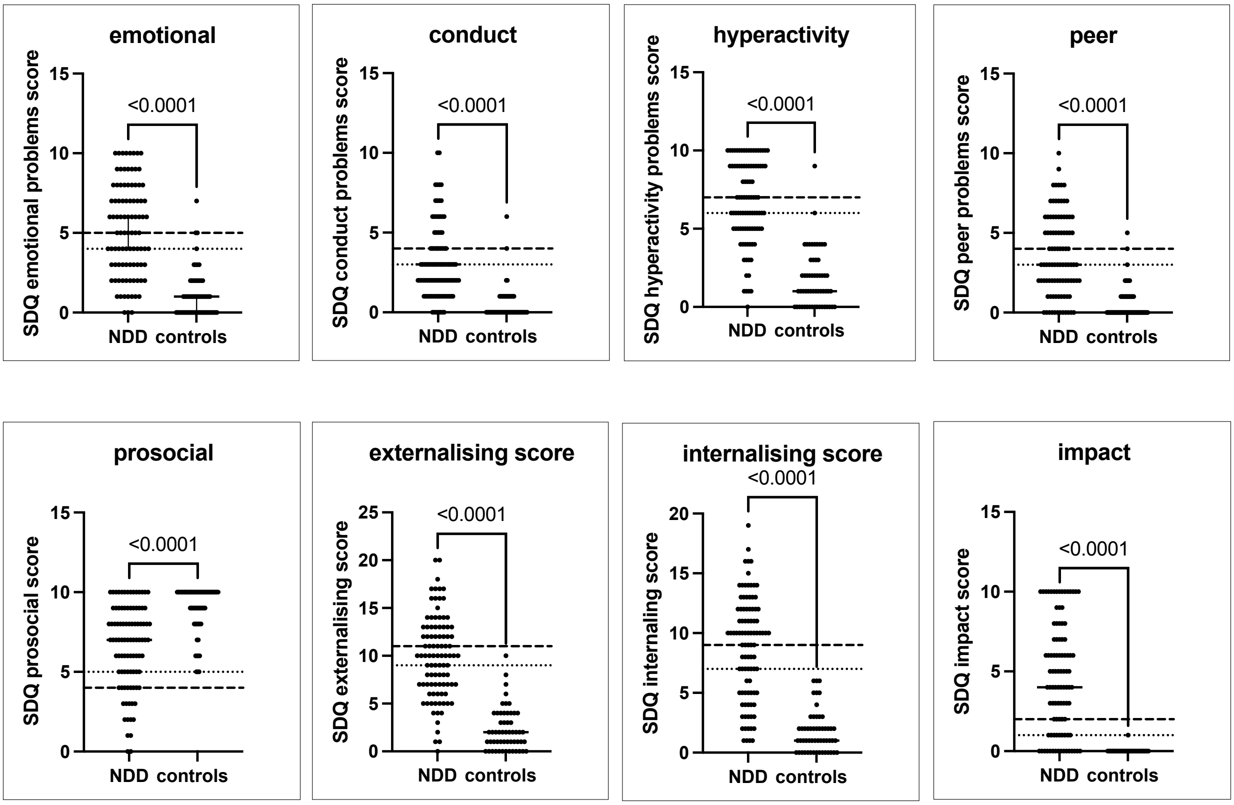

Supplementary Figure 4B: Heatmap of loss of skills between PANS versus controls and PANS versus NDD non-PANS patients

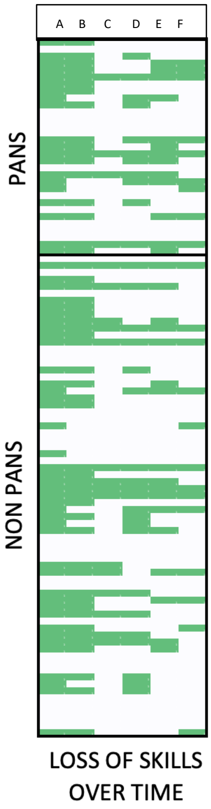

Heatmap of loss of skills between those who fulfil criteria for Paediatric acute neuropsychiatric syndrome (PANS- top half) and those who did not (NDD non-PANS- bottom half). On the x axis, A to F represent binary questions to loss of skills in 5 domains (A: Any loss of skills, B: Learning ability, C: language, D: Social skills, E: Fine motor, F: Gross motor skills).

Supplementary Figure 5A: Full blood count, C-reactive protein (CRP) and erythrocyte sedimentation rate (ESR) between PANS (n=20) and healthy controls (n=15) were not significant different between groups

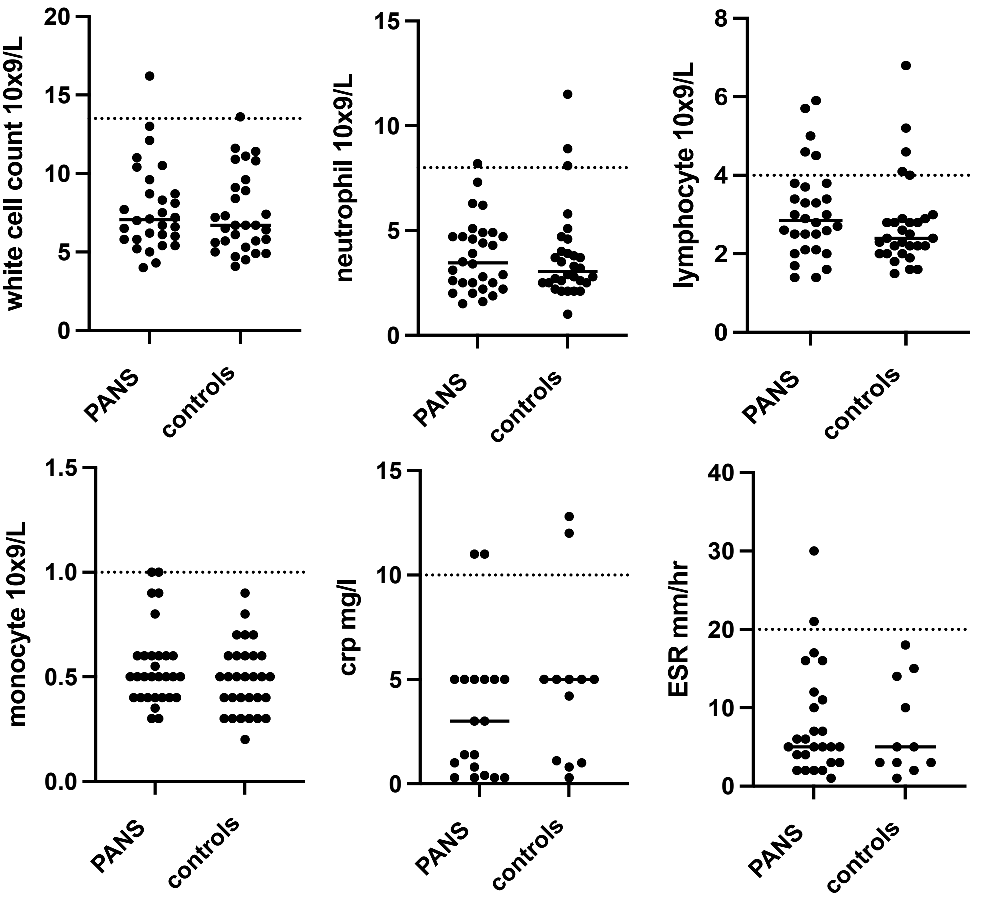

Supplementary Figure 5B: Full blood count between PANS-IVIg cohort (n=9) and healthy controls (n=10) were not significantly different between groups

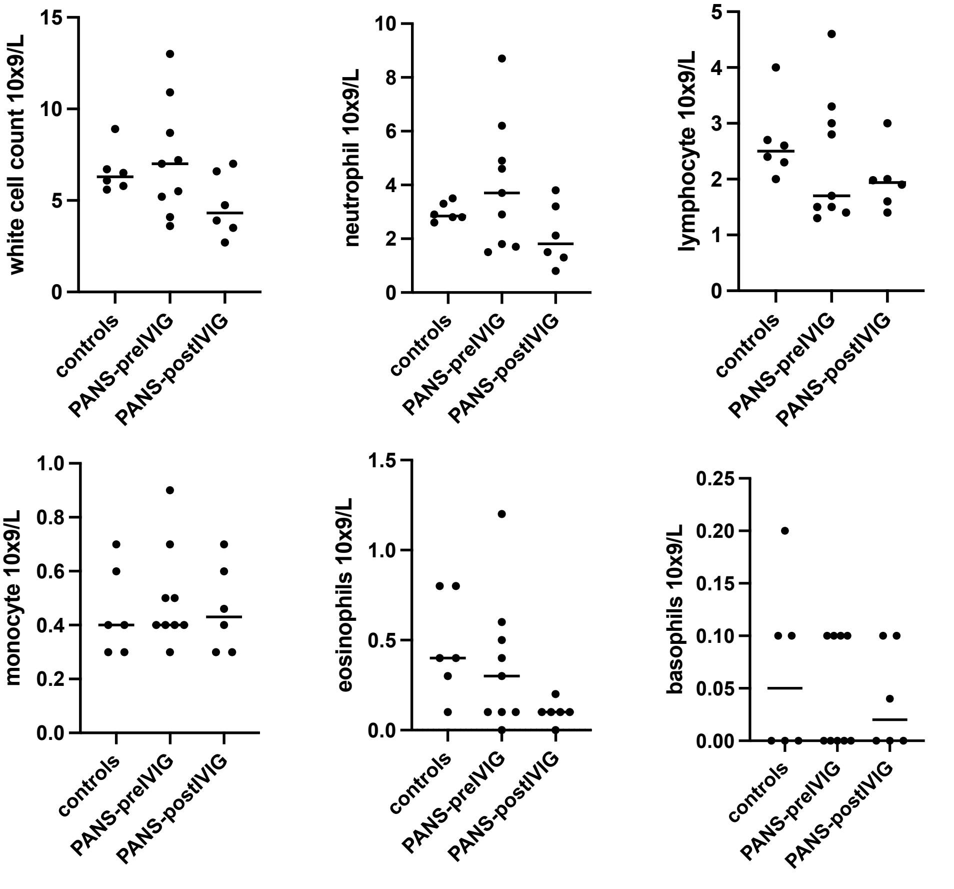

Supplementary Figure 6: PANS bulk RNA sequencing analysis

(A) Density of log count per million (logCPM) values of samples post normalization were similar across samples, reflecting technical reproducibility across samples.

(B) Removal of unwanted variation (RVU) canonical correlation plot is used to visualize the canonical correlation between factors of interest and gene expression. This graph shows how the canonical correlation changes with the number of singular vectors (*k*). In this study, *k=8* was used to remove genes that had minimal differential expression in all samples compared to negative control genes (on the left of the red dotted line).

(C) Box and whiskers relative log expression (RLE) plot of samples after normalization. (NDD= PANS patients, NC= normal controls)

(D) Heatmap of Pearson correlation coefficient between PANS (NDD= PANS patients) and NC (NC= normal controls). Pearson score of 1 (yellow) indicates high linear relationship between samples (ie. samples are more similar), while Pearson score less than 1 (dark blue) indicates higher difference between samples.

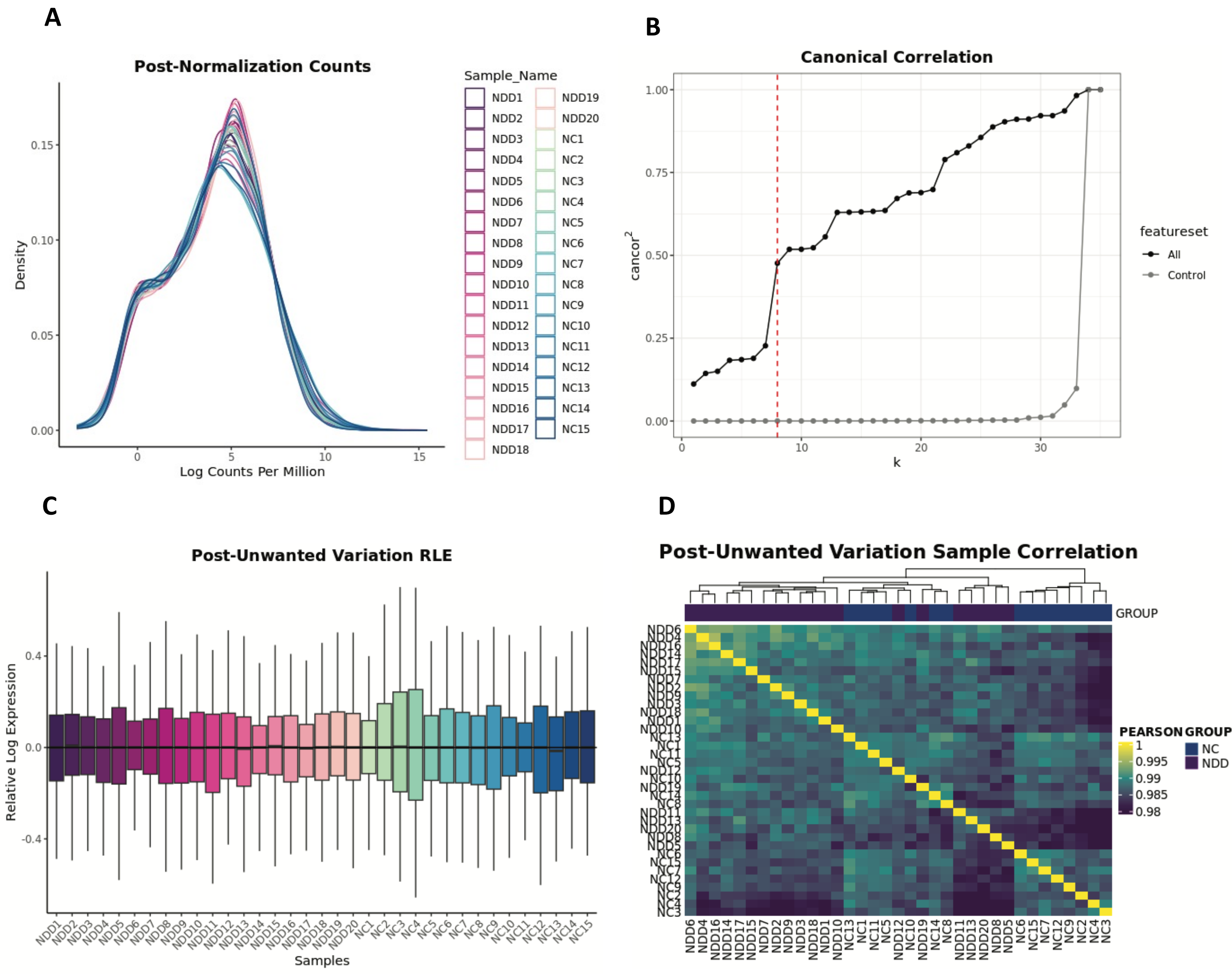

Supplementary Figure 7A: PANS bulk RNA sequencing analysis

Top 20 Gene Set Enrichment Analysis Gene Ontology pathways of (A) Biological process (BP) (B) Molecular function (MF) (C) Cellular component (CC) (D) Reactome of children with PANS compared to controls

Top 20 GSEA GO BP, MF, CC and reactome pathways

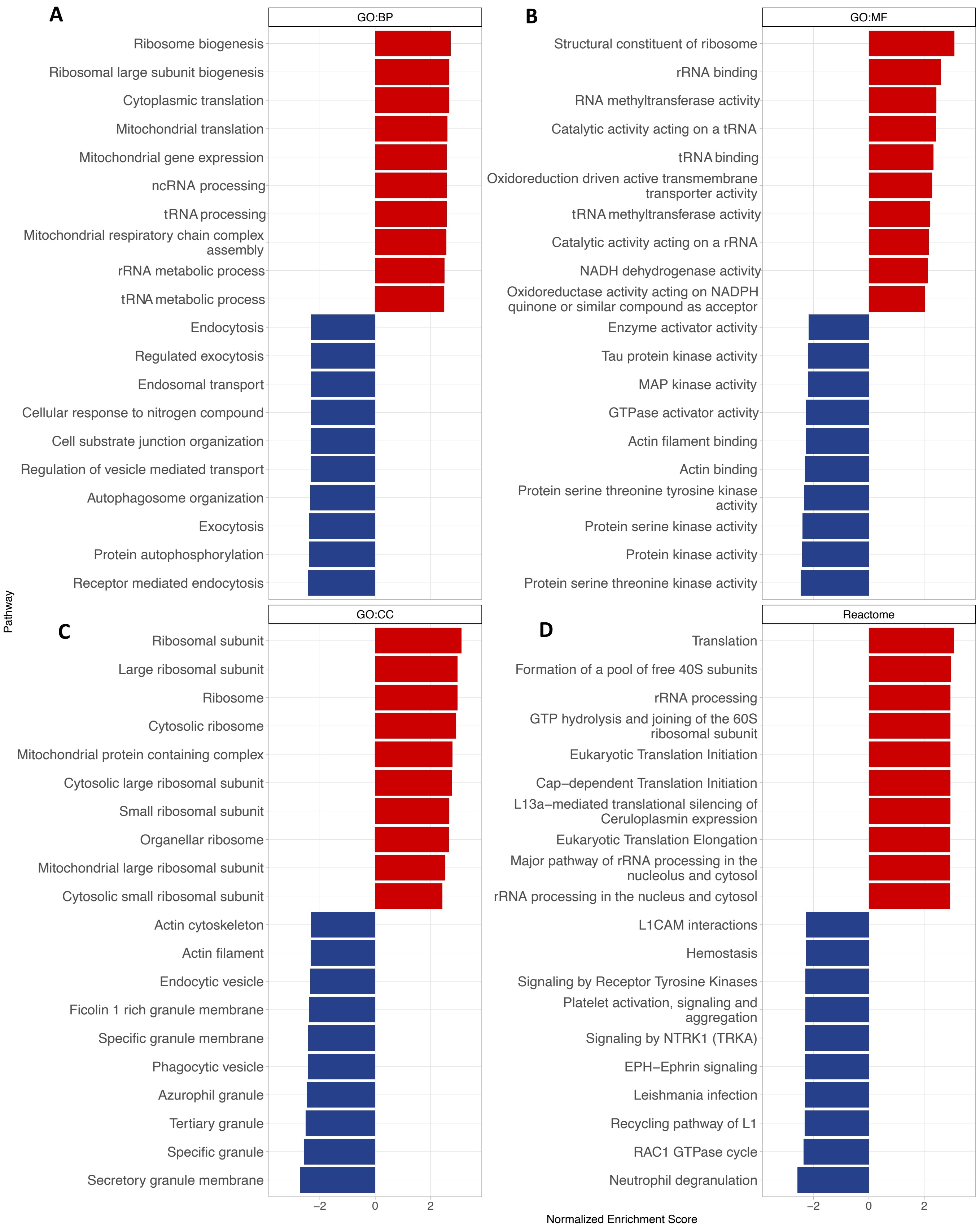

Supplementary Figure 7B: PANS bulk RNA sequencing analysis comparing
Top 10 Over representation analysis Gene Ontology A) PANS children not on neurotropic medications B) PANS children on neurotropic medications

There was no apparent effect of neurotropic medications on the bulk RNA sequencing results: children with PANS on neurotropic mediations (n = 9; mean age 10.4 years; 44.4% male) compared to age- and gender- matched healthy controls (n = 14; mean age 11.1 years; 35.7% male), had a similar RNA seq profile to children with PANS not on neurotropic medications (n = 11; mean age 7.1 years; 72.7% male).

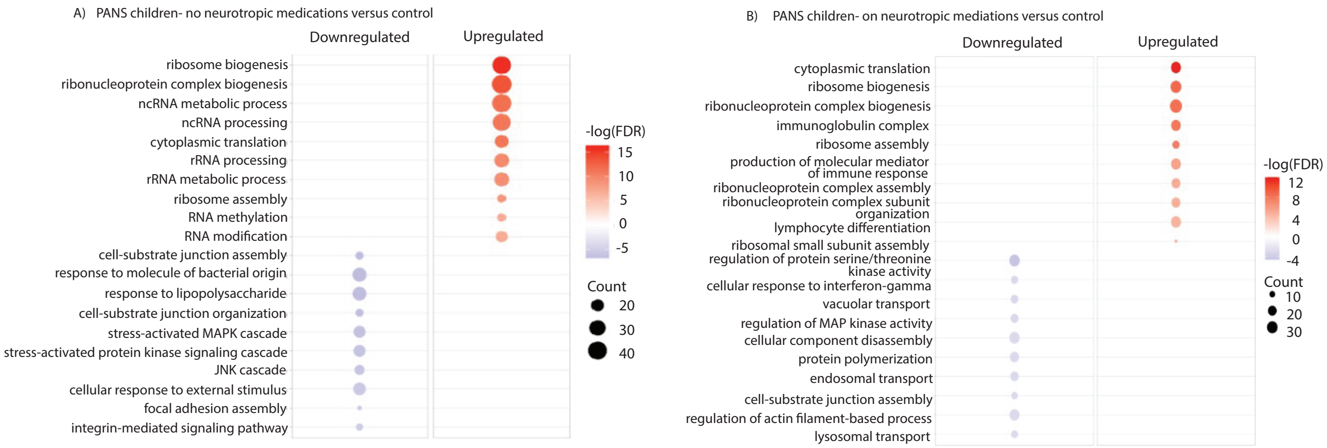

Neurotropic medications taken include Risperidone, Aripiprazole, Fluoxetine, Fluvoxamine, Sertraline, and Clonidine

Supplementary Figure 8: 10X single cell RNA sequencing analysis

(A) Mitochondrial profile of cells: Dot plot of proportion of mitochondrial genes counts within cells.

(B) Individual UMAP (Uniform Manifold Approximation and Projection) of 2 controls and 2 PANS patients did not show significant difference across 4 samples.

(C) Table of number of cells per population in the individual samples

A

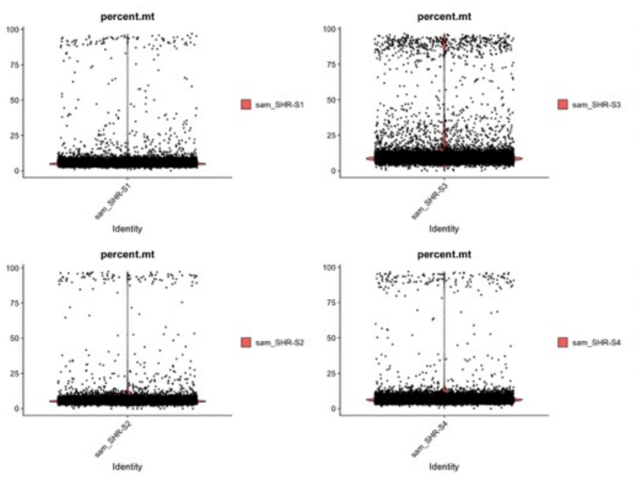

B

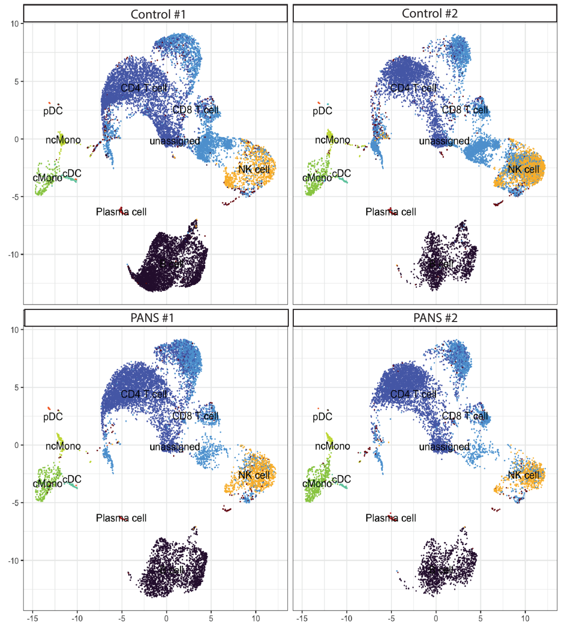

C

Populations Breakdown

condition	B cell	CD4 T cell	CD8 T cell	cDC	cMono	ncMono	NK cell	pDC	Plasma cell	unassigned
PANS1	1634	6073	2266	90	799	174	1089	20	43	224
Control1	1577	3103	4113	49	336	116	2328	4	26	279
PANS2	2142	4995	2217	39	293	76	868	17	16	163
Control2	4504	4692	2910	42	174	129	859	9	29	325

Supplementary Figure 9: PANS 10X single cell sequencing analysis.
Top 30 Gene Set Enrichment Analysis Gene Ontology pathways of (A) Biological Process (BP) (B) Molecular function (MF).

A Top 30 GSEA GO BP pathways

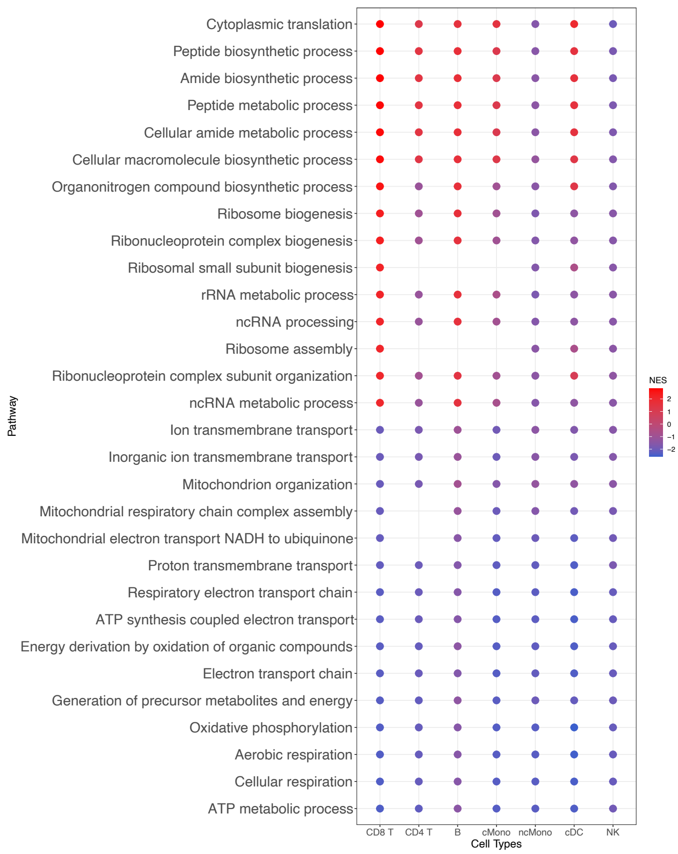

B Top 30 GSEA GO MF pathways

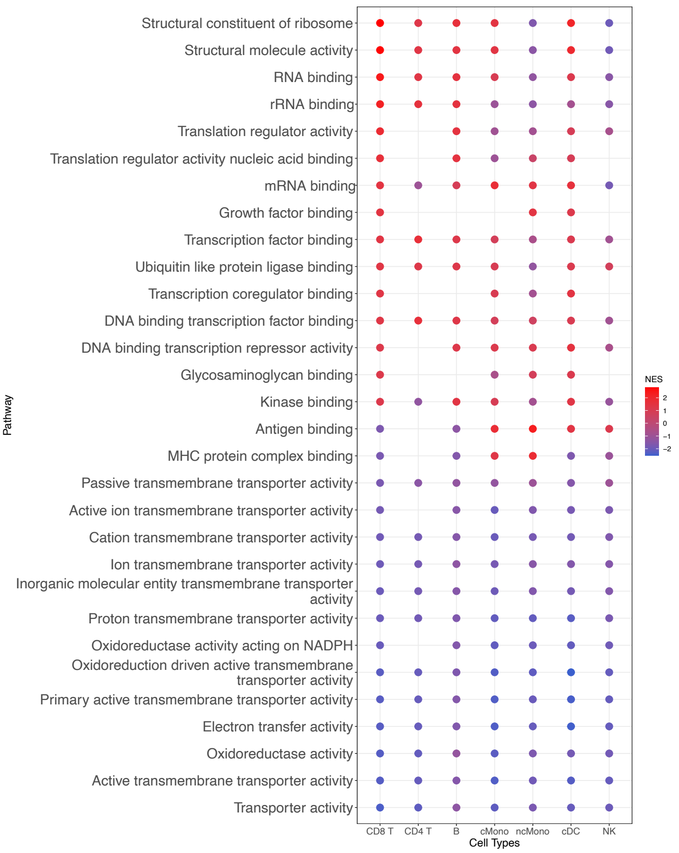

Supplementary Figure 10: PANS 10X single cell sequencing analysis.
Top 5 up and down regulated Gene Ontology Cellular Compartment (CC) pathways in specific cell types in children with PANS compared to controls

Top 10 GSEA GO CC pathways for all cell types

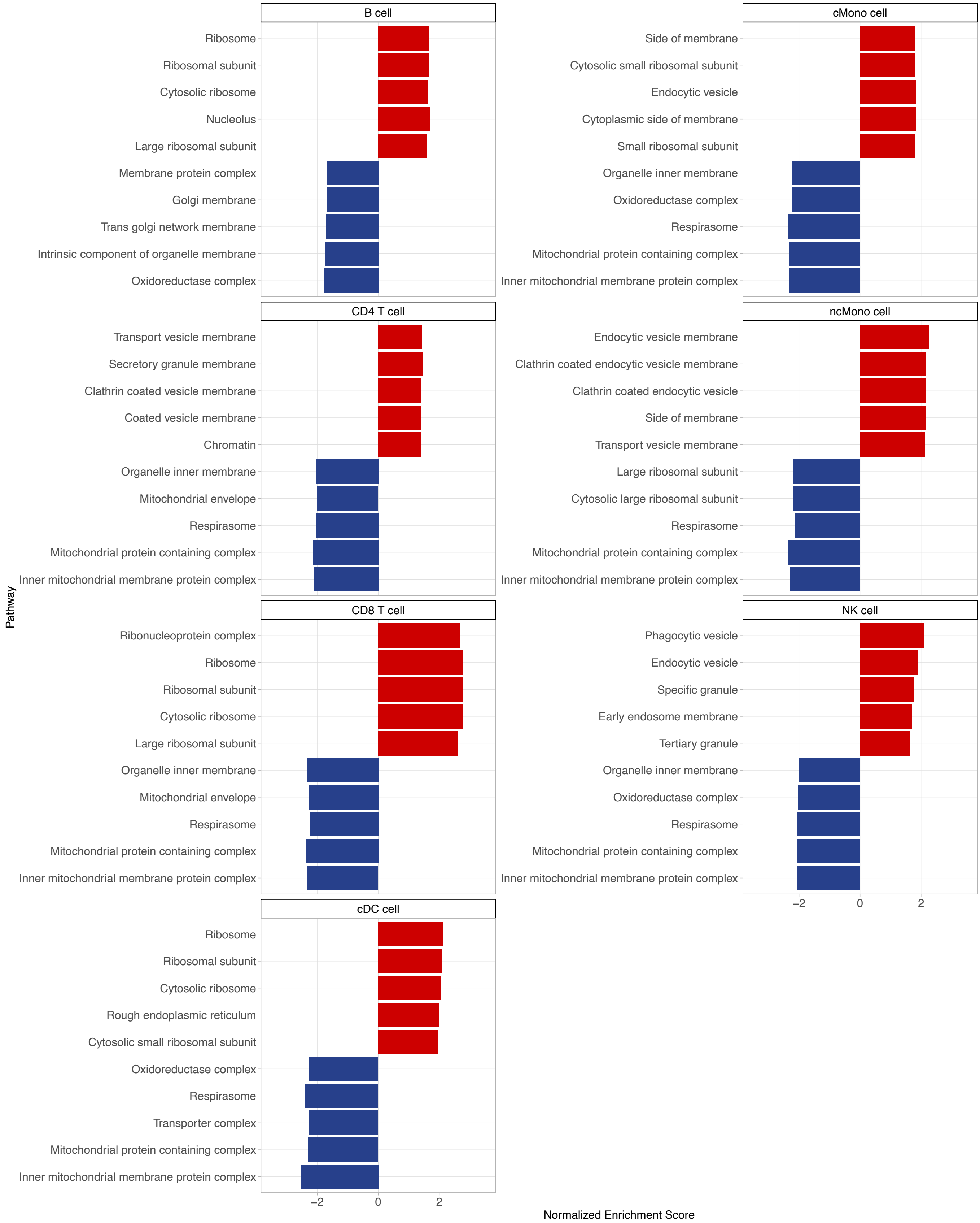

Supplementary Figure 11: PANS-IVIg bulk RNA sequencing analysis

(A) Removal of unwanted variation (RUV) canonical correlation plot is used to visualize the canonical correlation between factors of interest (*k*) and gene expression. In this study, *k=10* was used to remove genes that had minimal differential expression in all samples compared to negative control genes (on the left of the red dotted line).

(B) Box and whiskers relative log expression (RLE) plot of samples after normalization (PANS preIVIg (pre = PANS preIVIg batch 1, RNA_pre = PANS IVIg batch 2), PANS postIVIg (post = PANS postIVIg batch 1, RNA_post = PANS postIVIg batch 2) and NC (cont = control batch 1, RNA_cont = control batch 2)).

(C) Heatmap of Pearson correlation coefficient between PANS preIVIg (pre = PANS preIVIg batch 1, RNA_pre = PANS IVIg batch 2), PANS postIVIg (post = PANS postIVIg batch 1, RNA_post = PANS postIVIg batch 2) and NC (cont = control batch 1, RNA_cont = control batch 2). Pearson score of 1 (red) indicates high linear relationship between samples (ie. samples are more similar), while Pearson score less than 1 (dark blue) indicates higher difference between samples.

(D) Principal component analysis (PCA) performed on bulk RNA sequencing performed in PANS preIVIg (n=4), PANS postIVIg (n=4), and controls in cohort 1 (n=4). The x-axis represents Principal Component 1 (PC1), while the y-axis represents Principal Component 2 (PC2). Unbiased hierarchical clustering of gene expression between PANS preIVIg , PANS post IVIg and controls showed clustering in between groups.

(E) Principal component analysis (PCA) performed on bulk RNA sequencing performed in PANS preIVIg (n=5), PANS postIVIg (n=5), and controls (n=6) in cohort 2. The x-axis represents Principal Component 1 (PC1), while the y-axis represents Principal Component 2 (PC2). Unbiased hierarchical clustering of gene expression between PANS preIVIg , PANS post IVIg and controls showed clustering in between groups.

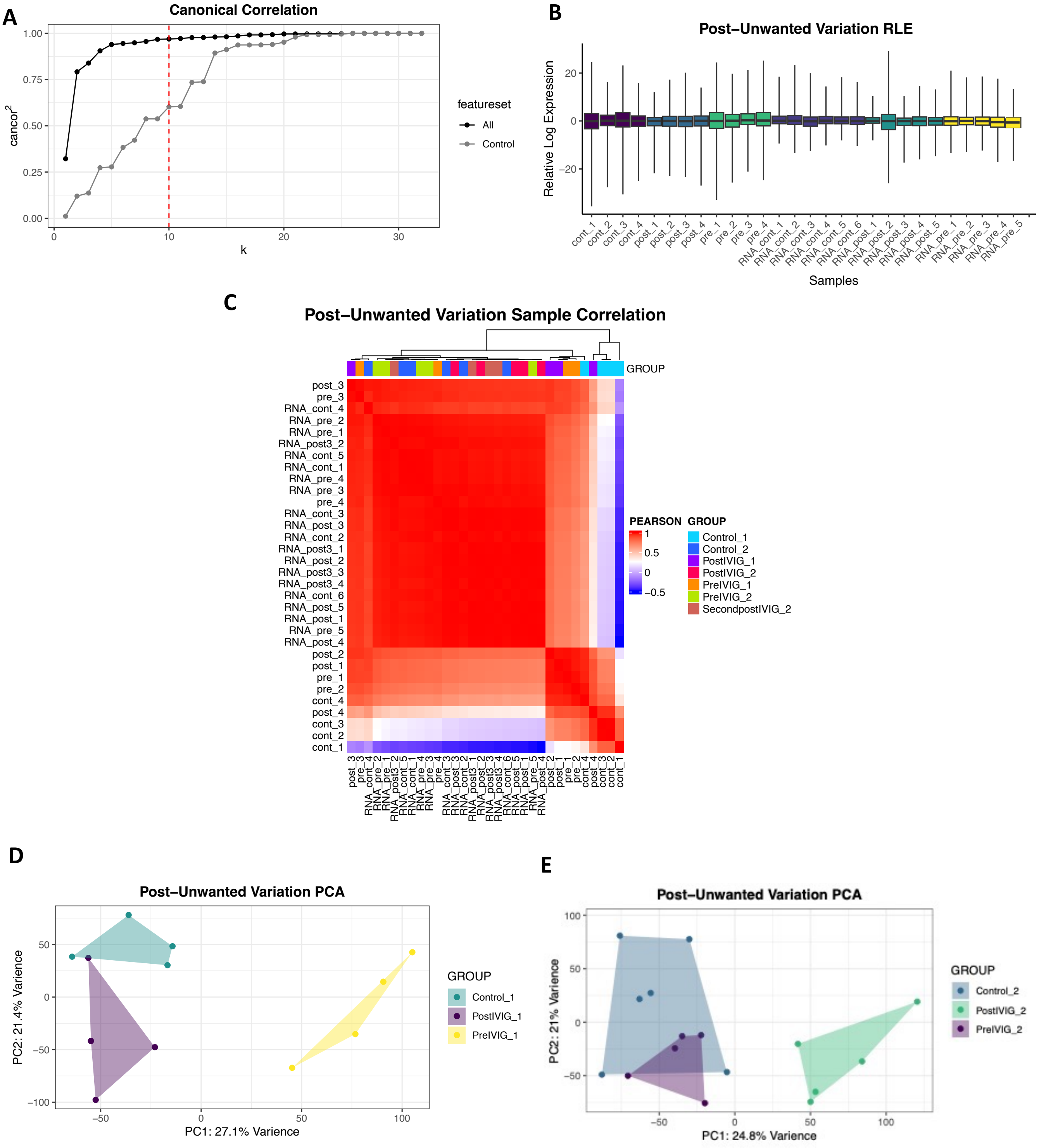

A
Canonical Correlation
cancor2
k
featureset
All
Control
1.00
0.75
0.50
0.25
0.00
0
10
20
30
B
Post−Unwanted Variation RLE
Relative Log Expression
Samples
20
0
−20
cont_1 cont_2 cont_3 cont_4 post_1 post_2 post_3 post_4 pre_1 pre_2 pre_3 pre_4 RNA_cont_1 RNA_cont_2 RNA_cont_3 RNA_cont_4 RNA_cont_5 RNA_cont_6 RNA_post_1 RNA_post_2 RNA_post_3 RNA_post_4 RNA_post_5 RNA_pre_1 RNA_pre_2 RNA_pre_3 RNA_pre_4 RNA_pre_5
C
Post−Unwanted Variation Sample Correlation
GROUP
post_3 pre_3 RNA_cont_4 RNA_pre_2 RNA_pre_1 RNA_post3_2 RNA_cont_5 RNA_cont_1 RNA_pre_4 RNA_pre_3 pre_4 RNA_cont_3 RNA_post_3 RNA_cont_2 RNA_post3_1 RNA_post_2 RNA_post3_3 RNA_post3_4 RNA_cont_6 RNA_post_5 RNA_post_1 RNA_pre_5 RNA_post_4 post_2 post_1 pre_1 pre_2 cont_4 post_4 cont_3 cont_2 cont_1
PEARSON
1
0.5
0
−0.5
GROUP
Control_1
Control_2
PostIVIG_1
PostIVIG_2
PreIVIG_1
PreIVIG_2
SecondpostIVIG_2
D
Post−Unwanted Variation PCA
PC2: 21.4% Varience
PC1: 27.1% Varience
50
0
−50
−100
−50
0
50
100
GROUP
Control_1
PostIVIG_1
PreIVIG_1
E
Post−Unwanted Variation PCA
PC2: 21% Varience
PC1: 24.8% Varience
100
50
0
−50
−50
0
50
100
GROUP
Control_2
PostIVIG_2
PreIVIG_2

Supplementary Figure 12: PANS-IVIg bulk RNA sequencing analysis

Top 5 up and down regulated Gene Ontology pathways of (a) Biological process (BP) (b) Cellular component (CC) (C) Molecular function (MF) (D) Reactome of children with Paediatric acute neuropsychiatric syndrome (PANS) at baseline compared to controls (left column) and post versus pre intravenous immunoglobulin treatment (right column)

Top 10 GSEA GO BP, CC, MF pathways

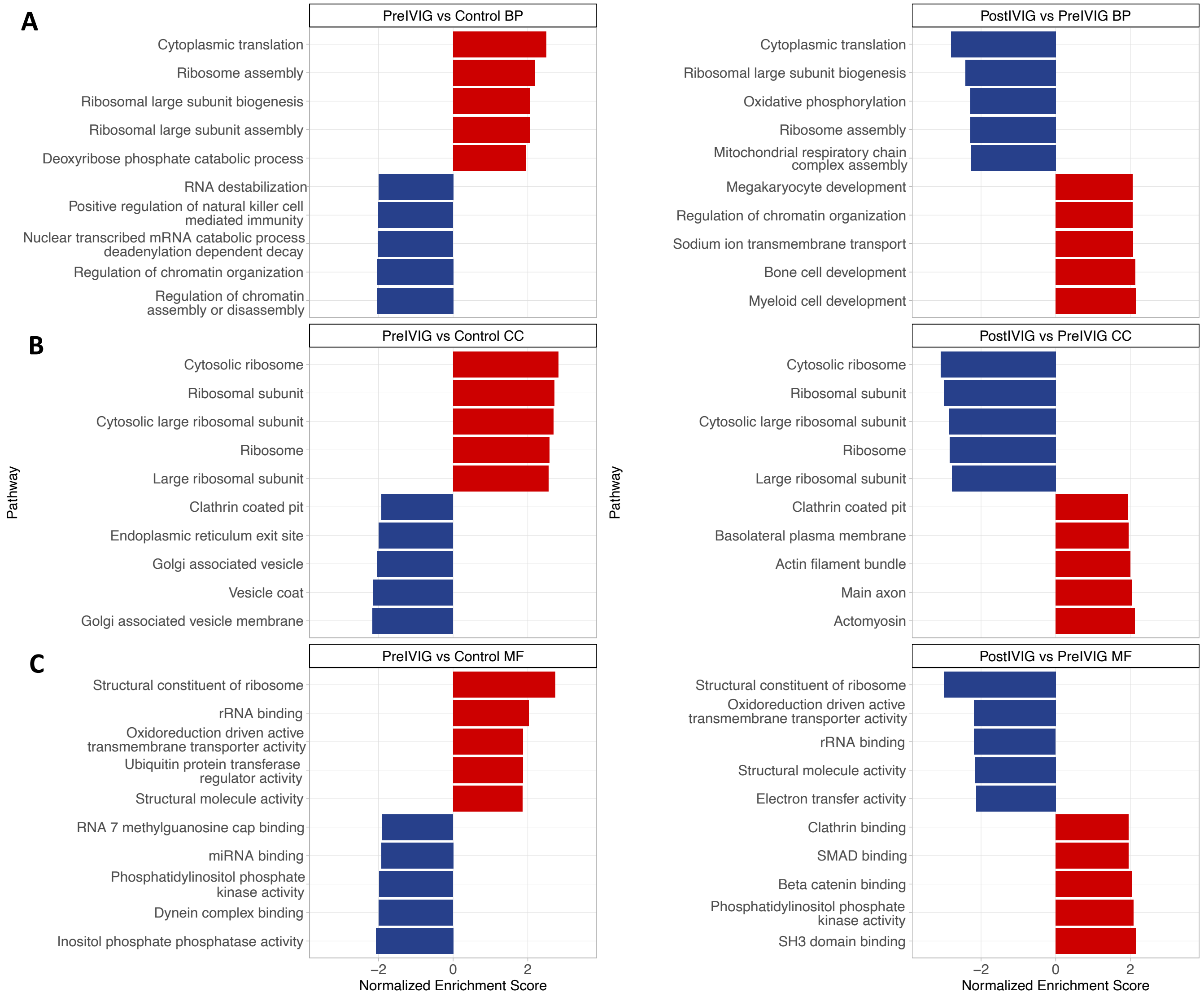

Top 10 GSEA reactome pathways

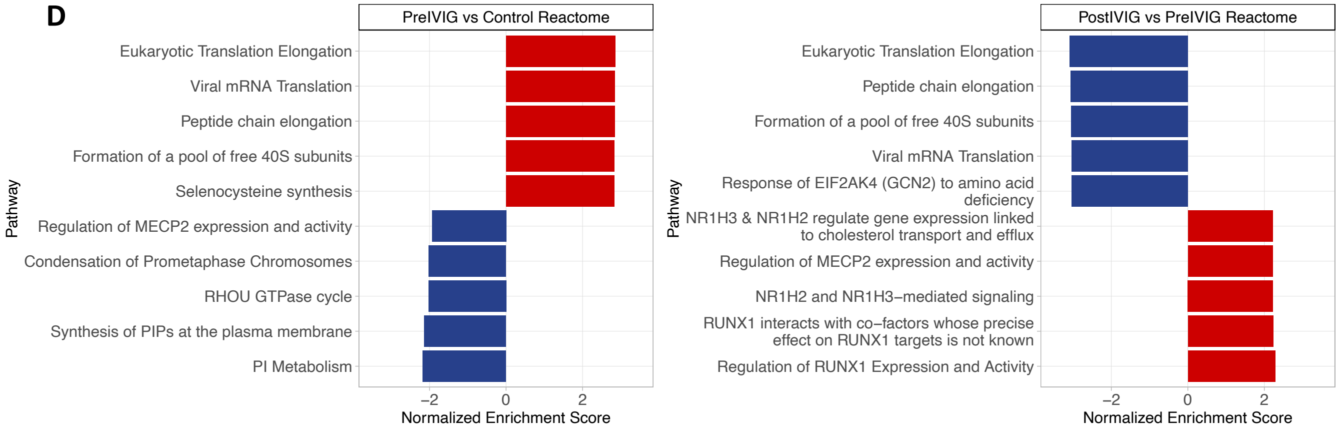

Supplement: Supplementary file 1 — Supplementary Material [file 41380_2025_3127_MOESM1_ESM.pdf]
